# Exploring the mechanism of active components from ginseng to manage diabetes mellitus based on network pharmacology and molecular docking

**DOI:** 10.1038/s41598-023-27540-4

**Published:** 2023-01-16

**Authors:** Ming-han Li, Ming-hui Jin, Rui-yi Hu, Shan Tang, Ke-ke Li, Xiao-Jie Gong, Yin-shi Sun, Ying-ping Wang, Zi Wang, Wei Li

**Affiliations:** 1 College of Chinese Medicinal Materials, Jilin Agricultural University, Changchun, 130118 China; 2 National and Local Joint Engineering Research Center for Ginseng Breeding and Development, Changchun, 130118 China; 3 Key Laboratory of Biotechnology and Bioresources Utilization, Ministry of Education, Dalian Minzu University, Dalian, 116600 Liaoning China; 4 Institute of Special Wild Economic Animals and Plants, Chinese Academy of Agricultural Sciences, Changchun, China

**Keywords:** Plant sciences, Systems biology

## Abstract

A large body of literature has shown that ginseng had a role in diabetes mellitus management. Ginsenosides are the main active components of ginseng. But what ginsenosides can manage in diabetic are not systematic. The targets of these ginsenosides are still incomplete. Our aim was to identify which ginsenosides can manage diabetes mellitus through network pharmacology and molecular docking. To identify the targets of these ginsenosides. In this work, we retrieved and screened ginsenosides and corresponding diabetes mellitus targets across multiple databases. PPI networks of the genes were constructed using STRING, and the core targets were screened out through topological analysis. Gene Ontology and Kyoto Encyclopedia of Genes and Genomes enrichment analyses were performed by using the R language. Finally, molecular docking was performed after bioinformatics analysis for verification. Our research results showed that 28 ginsenosides in ginseng might be against diabetes mellitus by modulating related proteins such as VEGFA, Caspase 3, and TNF-α. Among the 28 ginsenosides, 20(R)-Protopanaxatriol, 20(R)-Protopanaxadiol, and Ginsenoside Rg1 might play a significant role. Kyoto Encyclopedia of Genes and Genomes and Gene Ontology enrichment analysis showed that the management of diabetes mellitus by ginsenosides may be related to the positive regulation of reactive oxygen metabolic processes, associated with the insulin signaling pathway, TNF signaling pathway, and AMPK signaling pathway. Molecular docking results and molecular dynamics simulation showed that most ginsenosides could stably bind to the core target, mainly hydrogen bonding and hydrophobic bond. This study suggests the management of ginseng on diabetes mellitus. We believe that our results can contribute to the systematic study of the mechanism of ginsenosides for the management of diabetes mellitus. At the same time, it can provide a theoretical basis for subsequent studies on the management of ginsenosides in diabetes mellitus.

## Introduction

Diabetes mellitus (DM) is a non-communicable metabolic disease characterized by chronic hyperglycemia. It had become the third epidemic following cardiovascular diseases and tumors. Significant and persistent hyperglycemia can lead to dysfunction in various cell types, efficiently inducing complications such as nephropathy, retinopathy, angiocardiopathy, cerebrovascular diseases, and neuropathy^1^. More than 400 million people already had DM, and the number of people with the disease continues to increase worldwide^2^. According to the International DM Federation, there will be 700 million people with DM by 2045^3^. Although a growing number of researchers had discovered effective drugs that can manage DM, a complete and fundamental cure for type 2 DM remains difficult^4^. Currently, the drugs that can manage DM were mainly insulin or its peptide derivatives. In addition, dietary control can also contribute to the management of DM^5^. It is important to note that insulin and its peptide derivatives need to be injected intravenously, which can cause inconvenience to diabetic patients. More importantly, the long-term use of such chemical drugs may be harmful to the body of diabetic patients^6^. For these reasons, scholars have turned their attention to natural chemical-based substances with less toxic side effects, such as traditional herbal medicines. Excitingly, the management of DM by Chinese herbs has been clearly documented in classical Chinese medical writings^7^.

Ginseng (*Panax ginseng* C.A. Meyer) had a long history of use and was edible in China and had been widely recorded in various ancient texts of Chinese medicine^7^. In China, ginseng was used to treat Xiaoke disease, which begins with overeating and ends with dramatic weight loss. The clinical symptoms of this disease were almost identical to those of DM mellitus^8^. Studies have shown that steroidal structure is responsible for its multiple pharmacological activities, enabling ginsenosides to be absorbed to control biological activities at the transcriptional level^9^. Ginsenosides could manage the concomitant conditions of DM, such as anti-angiogenic, anti-apoptotic, and hepatoprotective^10,11^. However, research on which ginsenosides in ginseng play a role in managing DM is still incomplete. Which ginsenosides act on which targets regulate which signaling pathways and which biological processes (BP) are involved in managing DM have not been systematically reported. Importantly, it may be difficult to obtain ginsenosides in large quantities and with high purity but with low content in ginseng, which is not conducive to the conduct of experiments. Moreover, DM models often require a long modeling time in vivo experiments^12^. These make routine experiments face many difficulties.

Chinese herbal medicines involve multiple components and multiple targets and pathways in the process of managing diseases. This could lead to a more difficult exploration of mechanisms^13^. Therefore, it is appropriate, ingenious, and suitable to use network pharmacology to explore the mechanisms of herbal medicine in treating diseases^14^. Several studies had reported that the use of network pharmacology and molecular docking methods or dynamics simulation can successfully predict the components, targets, signaling pathways, and mechanisms of herbal medicines for the treatment of diseases. This method can save a lot of time and money. Moreover, it helped to explore the active ingredients and mechanism of action of herbal medicines^15,16^.

Therefore, through this work, we hope to obtain as much as possible the components that can play a role in managing DM and to clarify the mechanism by which ginsenosides manage DM.

## Methods and analysis

### Active ingredient screening and collection of diabetes mellitus relevant targets

First, we obtained the active ingredients and targets in ginseng through the database and analysis platform of Traditional Chinese Medicine Systems Pharmacology (TCMSP, https://www.tcmsp-e.com/tcmsp.php). We selected components with OB ≥ 30% and DL ≥ 0.18 as targets. The ginsenosides managed diabetic were then searched for from the reported papers to look for the active ingredients. However, the proven ginsenosides were not removed for the above qualification. The ginsenosides were downloaded SDF format files from the PubChem database (https://pubchem.ncbi.nlm.nih.gov). We used Swiss Target Prediction (http://www.swisstargetprediction.ch/) databases to obtain the targets of the ginsenosides. It be recorded when the predicted target probability > 0.1. Due to some ginsenosides had too many targets, such as ginsenoside 20(R)-Protopanaxatriol (PPT) had 94 targets. Too many targets will be detrimental to the subsequent analysis. Therefore, for ginsenosides with too many targets, we only selected the top 20 targets associated with DM for subsequent analysis. The UniProt database (https://www.uniprot.org/) was used to convert target protein names into target gene names to standardize gene names^17,18^.

Next, the GeneCards (GeneCards, https://www.genecards.org/), the Online Mendelian Inheritance of Human Beings (OMIM, https://www.omim.org/), the Pharmacogenetics and Pharmacogenomics Database (PharmGkb, https://www.pharmgkb.org), the Therapeutic Targets Database (TTD, http://db.idrblab.net/ttd/), and the Drugs and Drug Target Information Databases DM-related disease target genes (Drug Bank, https://go.drug/bank.com/) were integrated^18^. Finally, we used BioLadder (https://www.bioladder.cn/web/#/pro/index) to identify and visualise the intersecting targets of both ginsenosides and disease targets.

### Construction and analysis of the protein–protein interaction (PPI) network

We inputted the intersection targets into STRING 11.0 (https://string-db.org/) to obtain their reciprocal relationships^19^. Selection parameters were set to “Homo sapiens”, and the confidence level was set at 0.400 for the minimum required interaction score, and hid ligand disconnected nodes in the network. The PPI network was visualized by the Cytoscape 3.7.0 (https://cytoscape.org/) software. The network analyzer plugin in Cytoscape 3.7.0 was used to calculate topological parameters in the network. DC (Degree count) was used as the key indicator and BC (Betweenness centrality), CC (Closeness centrality), EC (Eigenvector centrality), LAC (Local Average Connectivity-based method), and NC (Network centrality) were used as secondary indicators to screen the core targets^20,21^. Finally, the key targets of the component ginsenosides against DM were obtained.

### Gene ontology and Kyoto encyclopedia of genes and genomes pathway enrichment analysis

All intersecting targets were analyzed for Gene Ontology (GO) and Kyoto Encyclopedia of Genes and Genomes (KEGG) enrichment by the Bioconductor cluster analyzer of R 4.0.2 (https://www.r-project.org/). The results were ranked according to the number of molecules in pathways with a cut-off value (*P* < 0.05)^18^. Plotting histograms of GO and KEGG analysis using R. We collected some significant and DM related targets. These targets are present in the top 15 BP or signaling pathways. We used OringPro 8.6 (https://www.originlab.com/software) to draw a component-target-BP or signaling pathway parallel graph to show their relationship to each other. At the same time, we obtained the signal pathway map from the KEGG database (https://www.kegg.jp/kegg/kegg1.html)22.

### Molecular docking of ligands with receptor

We obtained the structure of the target protein from the RCSB PDB database (https://www.rcsb.org/). The 2D structures of active substances from ginseng were sought by the PubChem Database^23^. The ChemBio Draw 3D (https://www.chemdraw.com.cn/ChemBio3D.html) and Autodock Tool was used to optimise the structure of ginsenosides and receptor proteins and to convert the two formats^24^. PyMol (https://pymol.org/2/) was used to optimize receptor proteins^25^. We used Chem3D software to minimize the energy of the ligand. We used Pymol software to remove water and increase hydrogen bonds in the acceptor protein. Finally, AutDockTools were used to draw the grid and unify. Autodock Vina was used for molecular docking to obtain the conformation with the lowest binding energy during docking. In addition, we used standard molecules and core targets for molecular docking and molecular dynamics analysis. The same binding pocket of protein molecules was used in the docking process. After, the lowest energy binding profile was visualized. Discovery Studio and PyMol software were used to visualize docking results^26^.

### Molecular dynamics simulation

After the docking studies were completed, the active ingredients with the good binding ability to the protein were further evaluated for their stability in the binding pocket using the amber18 software package to run 100 ns molecular dynamics of protein-compound conjugates. The ff14SB force field and the TIP3P water model were performed at a constant temperature and pressure and a periodic boundary condition. The force field of the micro-molecule was generated using the antechamber in AmberTools. During molecular dynamics simulation, the hydrogen bond involved was constrained using the LINCS algorithm with 2.0 fs integration time step, and then the Particle Mesh Ewald method was applied to calculate the electrostatic interactions with the cutoff value set to 1.2 nm. The non-bonding interaction cutoff was 10 Å and was updated every 10 steps. In addition, the V-rescale temperature coupling and Berendsen method were used to keep the temperature and pressure constant at 300 K and 1 bar, respectively, to perform 100 ps NVT and NPT equilibrium simulation. Molecular dynamics simulations of 100 ns were performed under NPT conditions^27^.

### Investigation of binding affinity using molecular mechanics Poisson–Boltzmann surface area (MM-PBSA)

The relative binding energy of a protein–ligand complex is popularly used in MD simulations and thermodynamic computations. MM-PBSA in combination with MD simulations is used to calculate the binding energy of protein and ligand complexes using the equation ΔG (Binding) = G (Complex)-G (Receptor)-G (Ligand) , where “G (complex) is total free energy of the ligand–protein complex, G (receptor) and G (ligand) are total free energies of the isolated protein and ligand in the solvent, respectively. The molecular mechanic’s potential energy plus the energy of solvation may be used to determine the total free energy of any of the three entities stated (ligand, receptor, or complex). We used the “amber-mmpbsa module” tool to perform binding energy calculations. The stable trajectory observed between the 90–100 ns was chosen for the binding energy calculation by selecting representative snapshots with an interval of 50 frames^28^.

## Results

### Active ingredients’ screening and collection of DM-relevant targets

**Figure 1:**
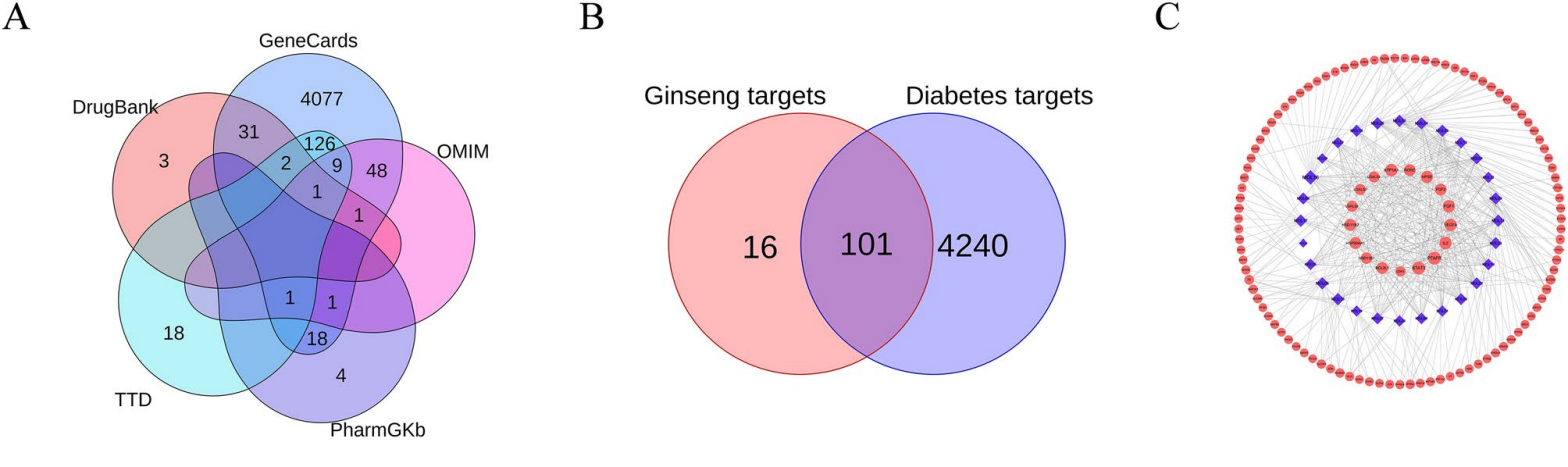
Ginsenoside-crosslinked targets visualization and core target screening. All disease targets DM (**A**). Ginsenosides-Disease intersection targets (**B**). Ginsenosides-intersection targets Network (**C**).

We obtained 28 ginsenosides through the TCMSP database combined with literature supplementation. These components and their information were presented in Table 1. After analyzing the data, we found that PPT, Ginsenoside Rg1 (Rg1), 20(S)-Ginsenoside Rh1 (Rh1), 20(S)-Ginsenoside Rh2 (Rh2), Ginsenoside Rh4 (Rh4), Ginsenoside Ro (Ro), and 20(R)-Protopanaxadiol (PPD) had more targets than other ginsenosides. Integrating five disease databases yielded 4341 DM-related targets as shown in Fig. 1A. The targets information of the ingredients and disease were in Supplementary Tables 1 and 2. The 101 intersection targets were obtained after integration, and a *Veen* diagram was drawn as shown in Fig. 1B. The specific intersection targets information were presented in Supplementary Table 3. Component-target network diagram for a more comprehensive elucidation of the mechanism of action of ginseng in DM by Cytoscape 3.7.0. In Fig. 1C, the blue diamond represents the components in the ginseng, and the pink ovals represent the targets. We found that ginsenoside PPT, Rg1, Rh1, Rh2, Rh4, Ro, and PPD had more targets for linkage compared with other ginsenosides. The results showed that each ginsenoside can act on multiple targets, and each target can also respond to multiple ginsenosides. This is consistent with the traditional Chinese medicine theory that the herbal treatment process is designed to be multi-component and multi-target.

**Table 1:** The ginsenosides and their information.

MOL ID	Composition	Molecular weight (g/mol)	Molecular formula
MOL01	20(R)-Ginsenoside Rg3	785.0	C_42_H_72_O_13_
MOL02	20(S)-Ginsenoside Rg3	785.0	C_42_H_72_O_13_
MOL03	20(R)-Panaxadiol	785.0	C_42_H_72_O_13_
MOL04	20(R)-Protopanaxatriol	476.7	C_30_H_52_O_4_
MOL05	20(R)-Ginsenoside Rg2	785.0	C_42_H_72_O_13_
MOL06	Ginsenoside C-K	622.9	C_36_H_62_O_8_
MOL07	Ginsenoside F2	785.0	C_42_H_72_O_13_
MOL08	Ginsenoside F4	767.0	C_42_H_70_O_12_
MOL09	Ginsenoside Rb2	1079.3	C_53_H_90_O_22_
MOL10	Ginsenoside Rc	1079.3	C_53_H_90_O_22_
MOL11	Ginsenoside Re	947.2	C_48_H_82_O_18_
MOL12	Ginsenoside Rf	801.0	C_42_H_72_O_14_
MOL13	Ginsenoside Rg1	801.0	C_42_H_72_O_14_
MOL14	20(S)-Ginsenoside Rg5	767.0	C_42_H_70_O_12_
MOL15	20(S)-Ginsenoside Rh1	638.9	C_36_H_62_O_9_
MOL16	20(S)-Ginsenoside Rh2	622.9	C_36_H_62_O_8_
MOL17	Ginsenoside Rh4	620.9	C_36_H_60_O_8_
MOL18	Ginsenoside Rk1	767.0	C_42_H_70_O_12_
MOL19	Ginsenoside Ro	957.1	C_48_H_76_O_19_
MOL20	Ginsenoside Rs1	1121.3	C_55_H_92_O_23_
MOL21	Ginsenoside Rs3	827.0	C_44_H_74_O_14_
MOL22	Ginsenoside Rb1	1109.3	C_54_H_92_O_23_
MOL23	Ginsenoside Rb3	1079.3	C_53_H_90_O_22_
MOL24	Ginsenoside Rd	963.2	C_48_H_82_O_19_
MOL25	Malonyl Ginsenoside Rd	1033.2	C_51_H_84_O_21_
MOL26	Malonyl Ginsenoside Rb1	1195.3	C_57_H_94_O_26_
MOL27	20(R)-Protopanaxadiol	460.7	C_30_H_52_O_3_
MOL28	Vina-Ginsenoside R4	963.2	C_48_H_82_O_19_

### Construction and analysis of the PPI network

**Figure 2:**
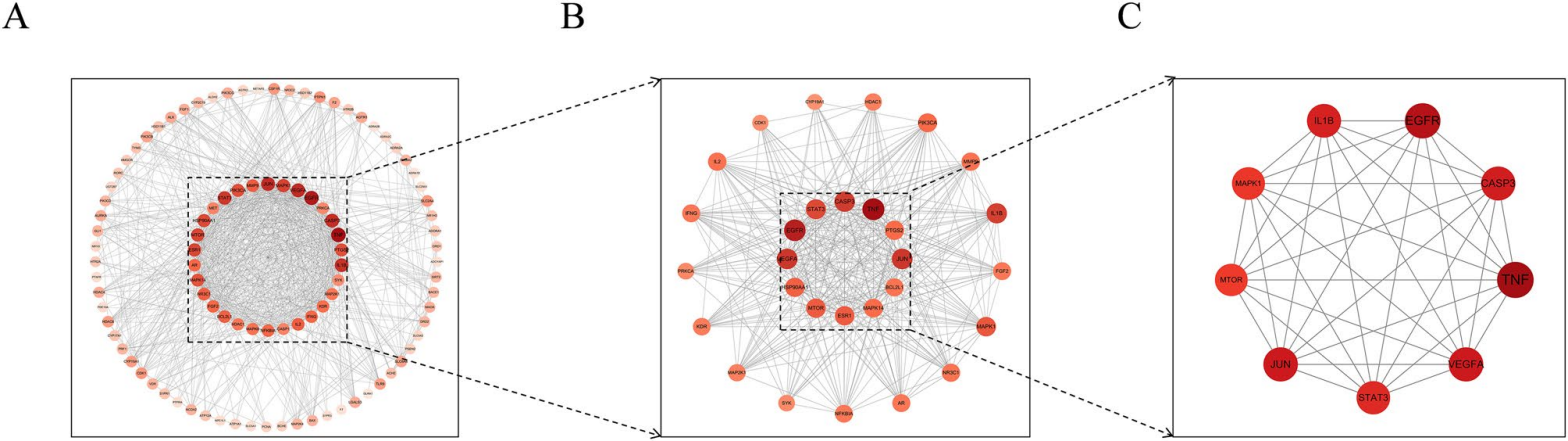
Protein–protein interaction network (**A**). The targets were obtained after the initial screening (**B**). The core targets were obtained after the final screening (**C**).

The core target network diagram was analyzed using Cytoscape 3.7.0. The results showed that 99 interacting target proteins were obtained by screening the String database. The core target network graph was composed of 99 nodes and 829 edges. Interactions were represented by each edge between proteins. The network analyzers identified 9 highly connected nodes (BC > 165.2336, CC > 0.5715, DC > 33, EC > 0.1695, LAC > 17.2749, and NC > 26.3852) as significantly core targets. A total of 99 protein–protein intersection targets were obtained and visualized in the PPI network (Fig. 2A). Figure 2B shown the minor core protein. As depicted in Fig. 2C, 9 core targets (IL-1β, EGFR, CASP3, TNF-α, VEGFA, STAT3, JUN, mTOR, EGRF, MAPK1) were screened out by sing Cytoscape 3.7.0. The names and details of the nine core targets were presented in Table 2. After comparison with existing reports, these targets played a major role in the development of DM.

**Table 2:** The 9 core targets of detailed information on the screening.

Name	BC	CC	DC	LAC	NC	EC
IL-1β	502.7261	0.6363	46	18.8260	35.7847	0.1917
mTOR	168.2533	0.5939	40	21.2000	32.5901	0.1889
Caspase 3	384.6253	0.6322	47	20.7659	39.3970	0.2035
VEGFA	326.1815	0.6405	48	21.3750	40.8360	0.2097
STAT3	218.9172	0.6125	44	21.8636	38.2908	0.2017
MAPK1	417.6774	0.6049	41	19.4146	31.8353	0.1830
JUN	270.7765	0.6322	48	21.8333	41.4560	0.2114
TNF-α	1048.993	0.6805	57	18.4912	47.9422	0.2139
EGFR	991.2846	0.6758	53	19.6603	43.5801	0.2109

*BC* Betweenness centrality, *CC* Closeness centrality, *DC* Degree count, *LAC* Local Average Connectivity-based, *NC* Network centrality, *EC* Eigenvector centrality.

### GO and KEGG pathway enrichment analysis

**Figure 3:**
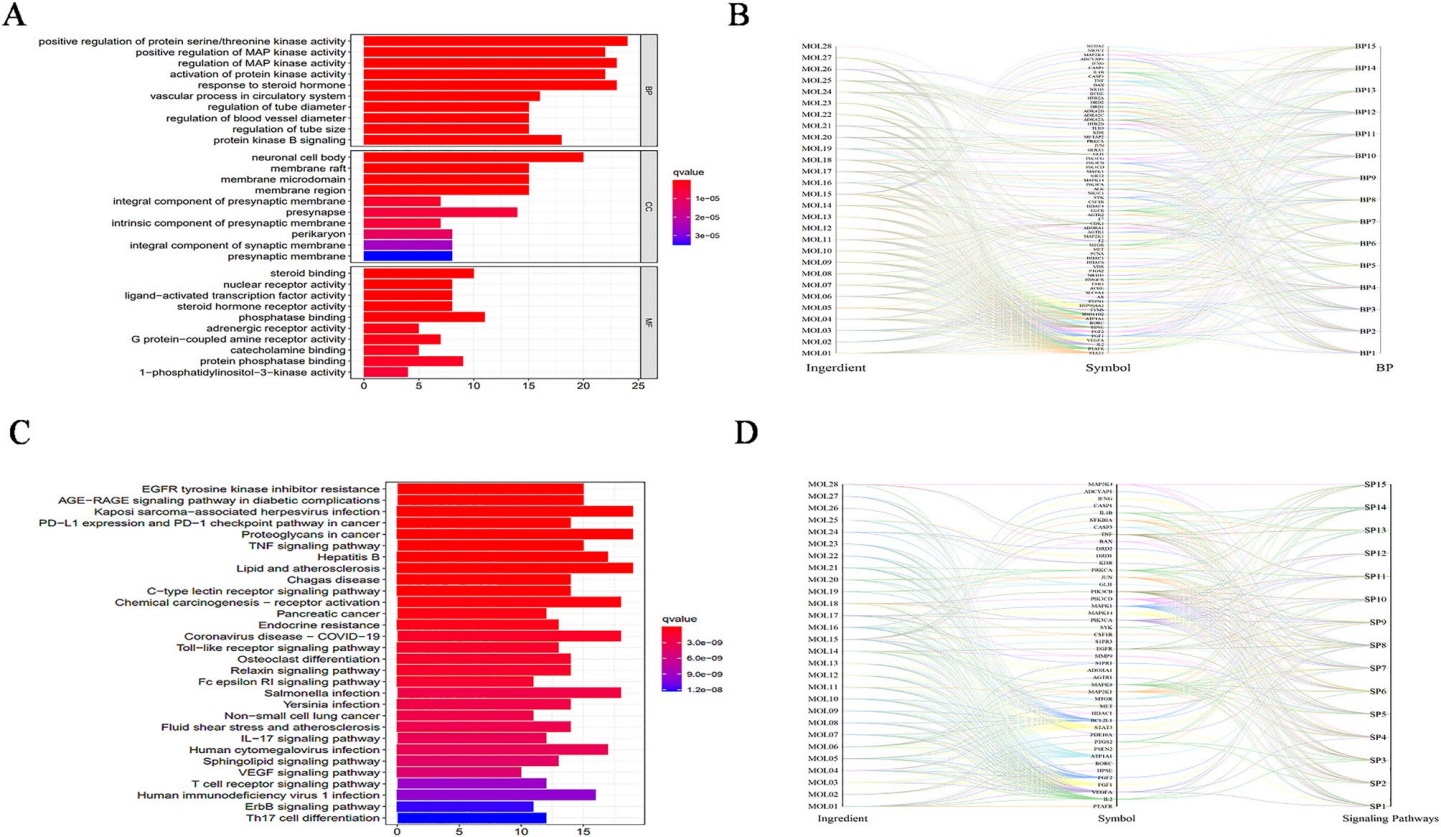
KEGG and GO enrichment analysis. GO enrichment analysis for all targets (**A**). Visualization of ginsenosides -target- pivotal biological process interactions. (**B**). KEGG enrichment analysis for all targets (**C**). Visualization of ginsenosides-target-signaling pathway interactions (**D**).

**Figure 4:**
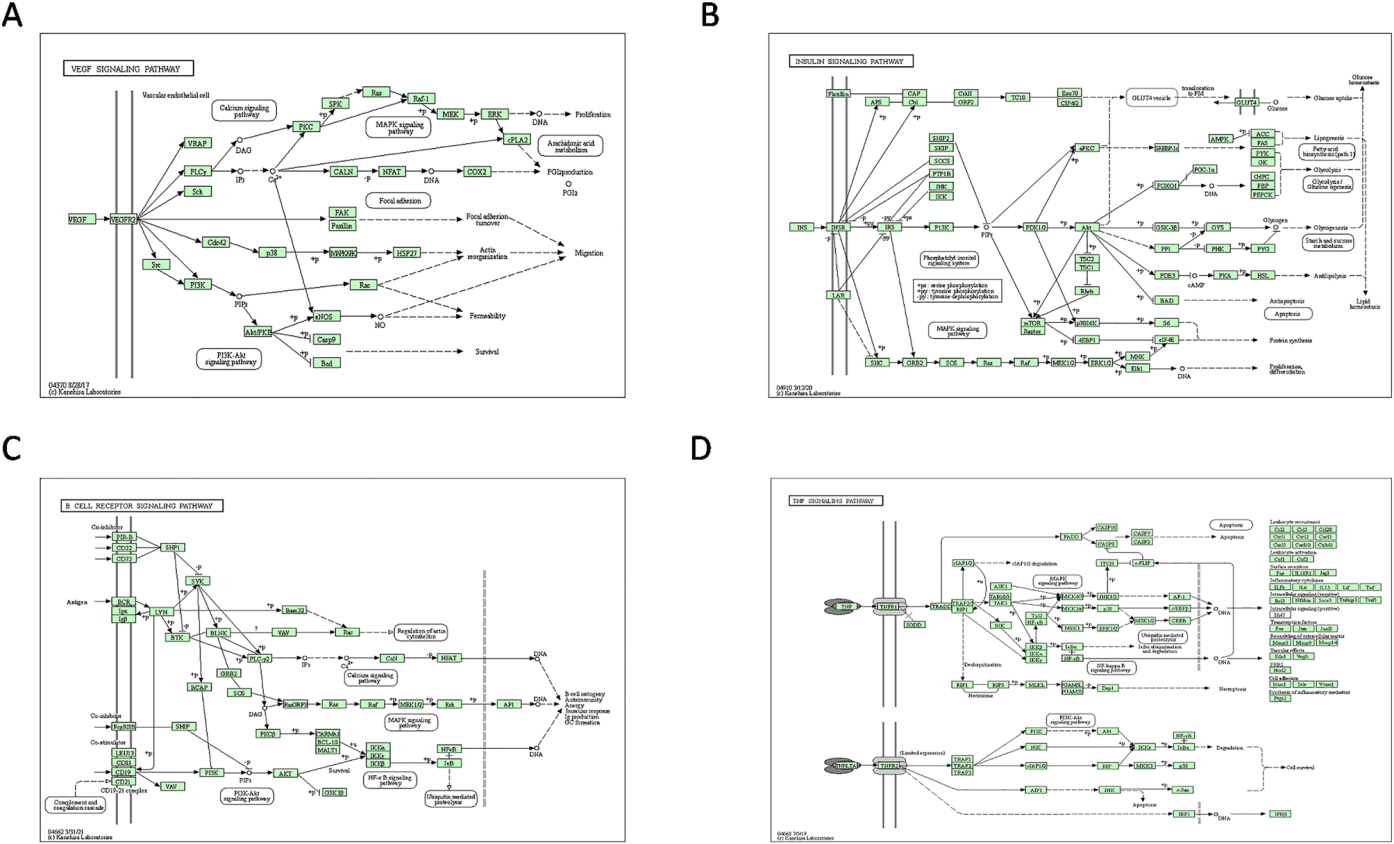
The four key signal path maps are from the KEGG database. (https://www.kegg.jp/kegg/kegg1.html). VEGF signaling pathway map (**A**). Insulin signaling pathway map (**B**). B cell receptor signaling pathway map (**C**). TNF signaling pathway map (**D**).

GO functional enrichment analysis of key targets was performed using the Bioconductor package in R software. Filter GO terms based on *P* values (*P* < 0.05, Q < 0.05). There were 2029, 56, and 153 GO terms associated with BP, cellular components, and molecular functions, respectively (Fig. 3A). As shown in Fig. 4A, the top 10 BP were related to positive regulation of protein serine/threonine kinase (STK) activity, positive regulation of MAP kinase activity, positive regulation of reactive oxygen species metabolic process, and regulation of lipid metabolic process, response to a steroid hormone, etc. In terms of cellular components, targets were enriched in the mitochondrial outer membrane, ATPase dependent transmembrane transport complex. Functional analysis showed steroid hormone receptor activity, protein STK activity, glucose transmembrane transporter activity, monosaccharide transmembrane transporter activity, etc. The component-target-BP network interaction diagram. From left to right in the picture were the ingredient information, target information, and BP information (Fig. 3B). The GO enrichment analysis information and 15 selected GO were provided in Supplementary Tables 4 and 5.

The KEGG involved 169 signaling pathways. These signaling pathways were the AGE-RAGE, AMPK, VEGF, Insulin, Adipocytokine, and PI3K-Akt signaling pathway (Fig. 3C). Figure 3D showed the component-target-signaling pathway network interaction diagram. From left to right in the picture were ingredient information, target information, and signaling pathway information. The KEGG enrichment analysis information and 15 selected KEGG were provided in Supplementary Tables 6 and 7. Combined with the previous reports, we speculated that VEGF, Insulin, B cell receptor, and TNF signaling pathway might play an important role in the management of DM by ginseng (Fig. 4). At the same time, we obtained an open access license for KEGG signaling pathway images by Kanehisa laboratories (Supplementary Fig. 1). Fifteen BPs and signaling pathways were involved in 71 and 43 targets in that order. From the reciprocal network in Figs. 4B and D, we can know that each ginsenoside can act on different targets and these target proteins had a presence in BP or signaling pathways. It indicated that ginsenosides could play a role in regulating BP or signaling pathways. The results of the component-target network, component-target-BP, or signaling pathway network showed that PPT, Rh1, Rh2, Rh4, Rg1, Ro, and PPD had more complex networks compared to other ginsenosides.

### Ginsenosides and core target molecular docking results

We treated the top 9 scoring targets with each of the 28 ginsenosides for molecular docking. The docking protein data were sequentially downloaded from the PDB database IL-1β, EGFR, Caspase 3, VEGFA, STAT3, mTOR, MAPK1, TNF-α and JUN PDB format files. The screened 28 ginsenosides were downloaded from the PubChem database. The download format was an SDF file. AutoDock software was used to molecularly dock small molecule active ligands to large molecule receptor proteins. The grid box size and center details’ information were in Supplementary Table 8. We used the AutoDock Vina software to calculate the energy binding of the components to the receptor protein (Table 3). Among all the molecular docking results, the maximum binding energy was − 6.4 kcal/mol, and the minimum binding energy was − 10 kcal/mol. Moreover, 89.2% of the binding energy results were less than − 7.0 kcal/mol. When a small molecule ligand binds to a large molecule protein, the lower the binding energy indicates the more stable the two were bound. In addition, by comparing the docking and MD results, we found that Ginsenoside Rg1, Ginsenoside Rh4 and Protopanaxatriol had lower binding energies and better stability than the standard receptor proteins. This result further demonstrates the ability of ginsenosides to manage in diabetes. (Supplementary Table 9 and Supplementary Fig. 2).

**Table 3:** Molecular docking binding energy of ligand with receptor (kJ/mol).

Ligand—Receptor	CASP3	EGFR	IL-1β	JUN	MAPK1	mTOR	STAT3	TNF-α	VEGFA
20(R)-Ginsenoside Rg3	− 7.7	− 9.8	− 7.3	− 6.3	− 8.7	− 7.2	− 7.7	− 7.6	− 8.9
20(S)-Ginsenoside Rg3	− 7.3	− 9.5	− 7.7	− 6.3	− 8.4	− 6.3	− 8.6	− 7.3	− 9.4
20(R)-Panaxadiol	− 8.3	− 8.4	− 7.2	− 7	− 8	− 7.3	− 7.6	− 7.2	− 7.7
20(R)-Protopanaxatriol	− 8.3	− 8.1	− 7	− 6.4	− 8	− 7.2	− 8.1	− 7.4	− 7.5
20(R)-Ginsenoside Rg2	− 8.2	− 8.8	− 7.3	− 6.5	− 7.5	− 8.1	− 7.6	− 7.3	− 8.3
Ginsenoside C-K	− 7.8	− 8.6	− 7.2	− 6.5	− 8	− 7	− 7.5	− 6.8	− 9
Ginsenoside F2	− 8	− 9.4	− 7	− 6.3	− 7.7	− 7.9	− 7.5	− 6.8	− 8.2
Ginsenoside F4	− 8.8	− 8.6	− 7.7	− 6.7	− 7.9	− 8.1	− 7.5	− 8	− 9.5
Ginsenoside Rb2	− 8.2	− 9	− 7.3	− 6.1	− 7.9	− 6.3	− 7.9	− 7.6	− 9.7
Ginsenoside Rc	− 8	− 9.1	− 7	− 6.3	− 7.1	− 7	− 8	− 7.2	− 9.7
Ginsenoside Re	− 8	− 8.2	− 7	− 6.7	− 7.6	− 7.3	− 7.5	− 6.9	− 9.5
Ginsenoside Rf	− 7.8	− 8.2	− 6.6	− 6.6	− 8.2	− 7.5	− 7.7	− 7.5	− 9
Ginsenoside Rg1	− 7.1	− 9	− 6.6	− 6	− 7.2	− 7.2	− 6.9	− 7.3	− 8.2
20(S)-Ginsenoside Rg5	− 8.2	− 9.7	− 7.8	− 6.6	− 8.9	− 7.6	− 8.5	− 7.8	− 9.1
20(S)-Ginsenoside Rh1	− 8.3	− 8.5	− 7.1	− 6.1	− 7.8	− 7.1	− 7.1	− 7.1	− 7.8
20(S)-Ginsenoside Rh2	− 9.2	− 9.2	− 7.3	− 6.1	− 8.1	− 7.2	− 8	− 7.7	− 8.2
Ginsenoside Rh4	− 8.3	− 8.9	− 7.4	− 6.4	− 7.8	− 7.4	− 7	− 7.2	− 8
Ginsenoside Rk1	− 8	− 8.7	− 7.9	− 6.5	− 8.7	− 7	− 8.5	− 7.6	− 8.1
Ginsenoside Ro	− 8	− 9.9	− 7.5	− 7.3	− 8.1	− 7.9	− 8.2	− 8	− 9.4
Ginsenoside Rs1	− 7.9	− 9.2	− 7.9	− 6.6	− 7.9	− 7.1	− 7.5	− 8	− 9.3
Ginsenoside Rs3	− 8	− 9.4	− 7.4	− 6.5	− 8.2	− 6.8	− 8.5	− 7.4	− 8.8
Ginsenoside Rb1	− 7.3	− 9.2	− 6.8	− 7	− 7.4	− 7.5	− 7.1	− 6.8	− 9.2
Ginsenoside Rb3	− 7.7	− 9	− 7.1	− 6.8	− 8.2	− 6.2	− 8.3	− 6.7	− 9.5
Ginsenoside Rd	− 7.6	− 8.7	− 6.9	− 6.8	− 7.2	− 6.6	− 8.6	− 6.6	− 10
Malonyl Ginsenoside Rd	− 7.5	− 8.8	− 7.3	− 6.9	− 7.5	− 7.2	− 7.8	− 7.4	− 8.6
Malonyl-Ginsenoside Rb1	− 8.6	− 8.9	− 6.9	− 5.9	− 7.7	− 7.2	− 8.3	− 8.4	− 9
20(R)-Protopanaxadiol	− 8.1	− 8.4	− 6.6	− 6.5	− 7.7	− 7.1	− 7.7	− 7.3	− 8.4
Vina-Ginsenoside R4	− 7.7	− 9.6	− 7.1	− 6.5	− 7.5	− 7.4	− 7.9	− 7.8	− 9.8

**Figure 5:**
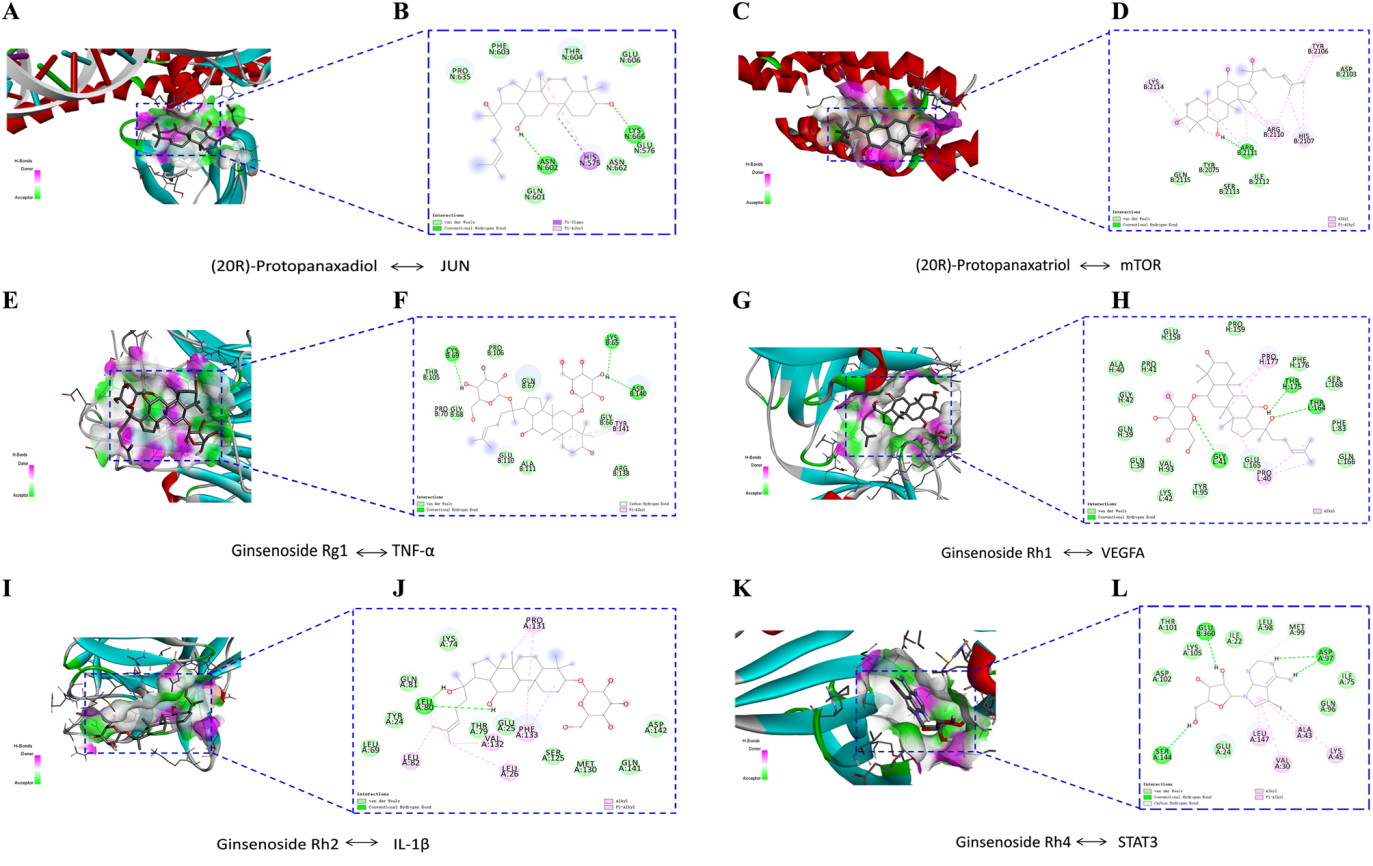
Molecular docking analysis showing bond pattern. 20(R)-Protopanaxadiol with JUN (**A**, **B**), 20(R)-Protopanaxatriol with mTOR (**C**, **D**), Ginsenoside Rg1 with TNF-α, (**E**, **F**), Ginsenoside Rh1 with VEGFA (**G**, **H**), Ginsenoside Rh2 with IL-1β (**I**, **J**), Ginsenoside Rh4 STAT3 (**K**, **L**). Areas of the donor and acceptor of hydrogen bond (H-bond) (**A**, **C**, **E**, **G**, **I**, **K**). 2D patterns of bond (**B**, **D**, **F**, **H**, **J**, **L**).

Some of the results were visualized with Discovery Studio and PyMol software. Figure 5A, B shown the interaction modes of ginsenosides with JUN receptor protein: PPD interacts with the residues ANS 602, and LYS 666 using hydrogen bonding, with the residue GLN 601, PRO 635, PHE 603, THR 604, GLU 606, GLU 576, ASN 662 via van der Waals interactions forces, with the residue HIS 575 via hydrophobicity in the form of a π bond interactions forces, and with the residue HIS 575 through a π Sigma effect. This process involved two hydrogen bonds and two hydrophobic bonds. The interaction modes of PPT with mTOR receptor protein: PPD interacts with the residues GLN 2115, TYR 2075, SER 2113, ILE 2112, ASP 2103 by way of van der Waals interactions forces, with the residues LYS 2114, ARG 2110, HIS 2107, TYR 2106 ARG 2110 via hydrophobicity and hydrophobicity in the form of a π bond interactions forces, with the residues ARG 2110 by means of a conventional hydrogen bond. One hydrogen bond and twelve hydrophobic bonds were involved in this process (Fig. 5C, D). The interaction modes of ginsenoside Rg1 with TNFα receptor protein: ginsenoside Rg1 interacts with the residues CYS 69, LYS 65, ASP 140 by means of hydrogen bonding, with the residues ALA 111, GLU 110, GLY 68, PRO 70, THR 105, PRO 106, GLN 67, GLY 66 via van der Waals interactions forces, with the residues PRO 70 via a carbon-hydrogen bond, with the residueTYR 141 via hydrophobicity in the form of a π bond interactions forces. This process involved four hydrogen bonds and two hydrophobic bonds (Fig. 5E, F). The interaction modes of ginsenoside Rh1 with VEGFA receptor protein: ginsenoside Rh1 interacts with the residues TYR 95, LYS 42, VAL 93, GLN 38, GLN 39, GLY 42, ALA 40, PRO 41, GLU 158, PRO 159, PHR 176, SER 168, PHE 83, GLN 166 by van der Waals interactions forces, with the residues GIY 41, THR 175, THR 164 by means of hydrogen bonding, with the residues PRO 177, PRO 40 via hydrophobicity. This process involved three hydrogen bonds and five hydrophobic bonds (Fig. 5G, H). The interaction modes of ginsenoside Rh2 with IL-1β receptor protein: ginsenoside Rh2 interacts with the residues LYS 74, GLN 81, TYR 24, LEU 69, THR 79, GLU 25, SER 125, MET 130, GLN 141, ASP 142 through van der Waals interactions forces, with the residues LEU 80 by means of hydrogen bonding, with the residues PRO 131, PHE 133, VAL 132, LEU 26, LEU 82, LEU 80 via hydrophobicity and hydrophobicity in the form of a π bond interactions forces. This process involved two hydrogen bonds and eight hydrophobic bonds (Fig. 5I, J). The interaction modes of ginsenoside Rh4 with STAT3 receptor protein: ginsenoside Rh4 interacts with the residues THR 101, LYS 105, ASP 102, GIUU 24, ILE 22, LEU 98, MET 99, ILE 75, GLN 96 by way of van der Waals interactions forces, with the residues GIU 360, ASP 97, SER 144 through hydrogen bonding, with the residues MET 99 via a carbon-hydrogen bond, with the residues LEU 147, ALA 43, VAL 30, LYS 45 by way of hydrophobicity and hydrophobicity in the form of a π bond interactions forces. This process involved five hydrogen bonds and four hydrophobic bonds (Fig. 5K, L).

### The dynamics stability simulation of ginsenosides with core receptor proteins

**Figure 6:**
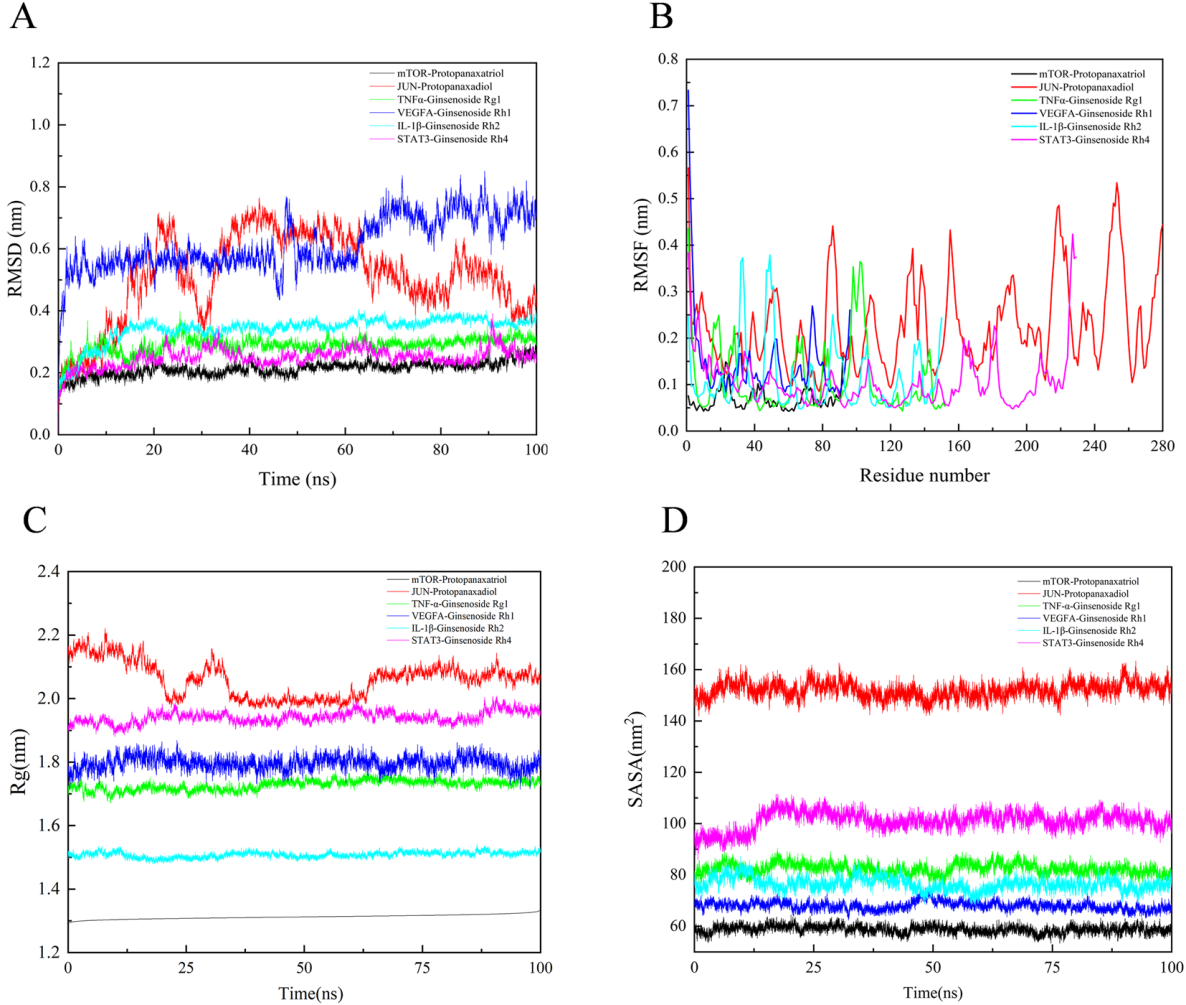
Profiles of molecular dynamics simulations. RMSD analysis for the protein–ligand complexes (**A**). RMSF analysis for the protein–ligand complexes (**B**). Rg analysis for the protein–ligand complexes (**C**). SASA analysis for the protein–ligand complexes (**D**).

We selected ginsenosides with more DM-related targets, and the selected receptor proteins were at the core of the network analysis. On this basis, we carry out the following molecular dynamics analysis. The ginsenosides—receptor protein was further selected to perform a computer molecular dynamics simulation study in the hope of understanding their stability in the binding pocket. Of note, the root means square deviation (RMSD) was an important basis to measure whether the system was stable or not. The mTOR-Protopanaxatriol, STAT3-Ginsenoside Rh4, TNFα-Ginsenoside Rg1, and IL-1β-Ginsenoside Rh2 systems were consistently lower fluctuation throughout the molecular dynamics simulations. Their average RMSD value were 0.21299 ± 0.00016 nm, 0.24917 ± 0.00021 nm, 0.28859 ± 0.00019 nm, and 0.34124 ± 0.00030 nm. The JUN-Protopanaxadiol and VEGFA-Ginsenoside Rh1 systems fluctuated slightly throughout the molecular dynamics simulations. Their average RMSD value were 0.65815 ± 0.00115 nm. The protopanaxadiol was unstable against JUN. But may be suitable drug candidate for JUN. (Fig. 6A). Next, the flexible change in amino acid residues in receptor protein and the root mean square fluctuation (RMSF) value were evaluated. We sequentially evaluated the average RMSF of mTOR-Protopanaxatriol, STAT3-Ginsenoside Rh4, TNFα-Ginsenoside Rg1, IL-1β-Ginsenoside Rh2, JUN-Protopanaxadiol, and VEGFA-Ginsenoside Rh1 system. Their average RMSF value were 0.06883 ± 0.00017 nm, 0.10717 ± 0.0039 nm, 0.10926 ± 0.00621 nm, 0.11252 ± 0.00580 nm, 0.22683 ± 0.00565 nm, and 0.14594 ± 0.00923 nm. The fluctuation frequency of the above six systems is low. Among them, the mTOR-Protopanaxatriol system had the least fluctuation (Fig. 6B). Taken together, all the results confirmed the stability of ginsenosides and core receptor proteins during molecular dynamics simulation.

**Figure 7:**
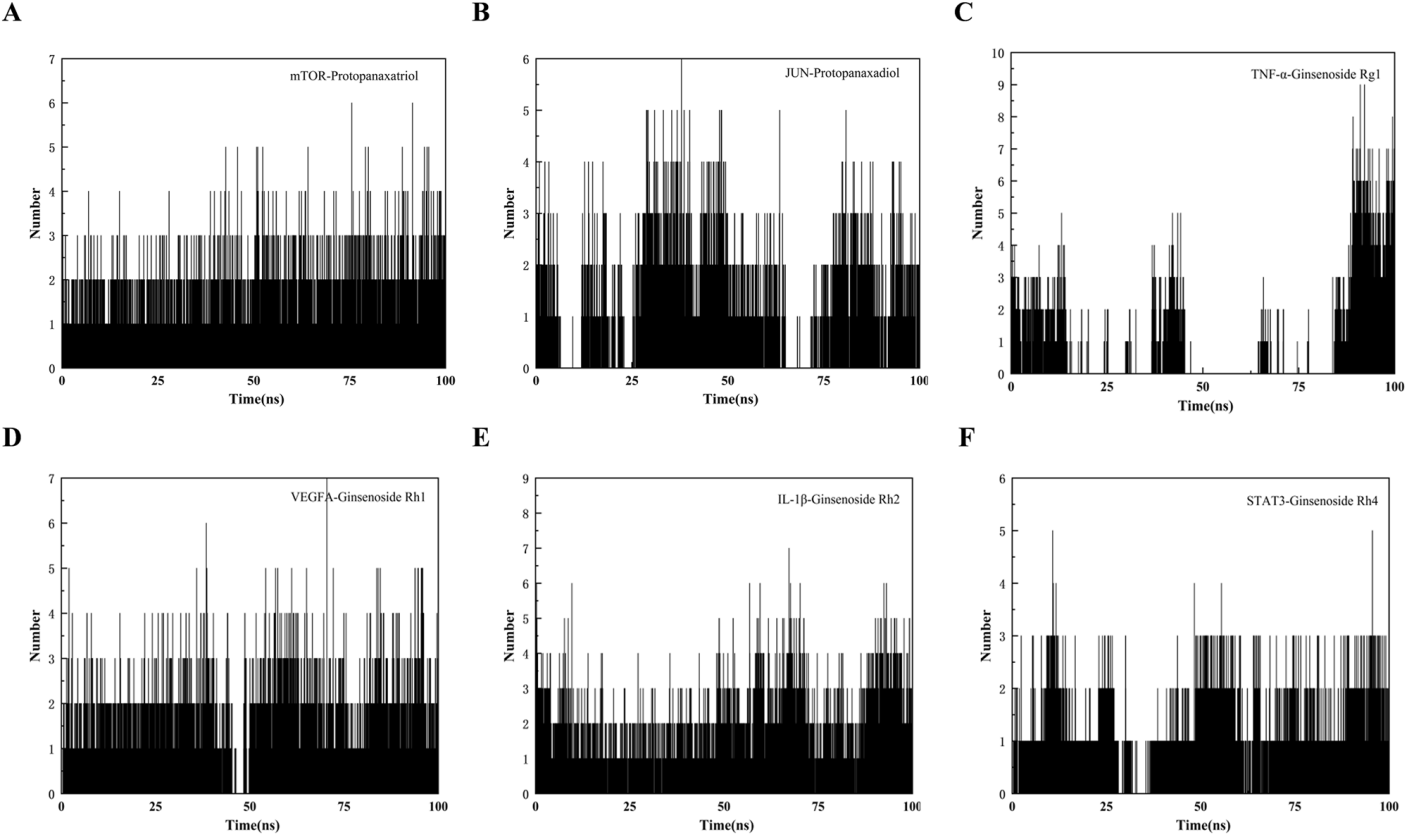
The number of hydrogen bonds involved in the binding of proteins with molecules during molecular dynamics simulations.

Among the six systems, mTOR-Protopanaxatriol and IL-1β-Ginsenoside Rh2 had the lowest mean Rg values of 1.31215 ± 0.00683 nm and 1.50815 ± 0.00893 nm respectively indicating that these two systems were the most stable. TNF-α—Ginsenoside Rg1, VEGFA-Ginsenoside Rh1, and STAT3-Ginsenoside Rh4 systems were more stable with Rg values of 1.72798 ± 0.015499 nm, 1.79617 ± 0.02037 nm, and 1.94129 ± 0.01815 nm. Jun- Protopanaxadiol was the most fluctuating among the six systems, and its Rg value of 2.03092 ± 0.05526 nm was more unstable (Fig. 6C). The solvent-accessible surface area (SASA) was analyzed to distinguish the protein compactness behavior. The results showed that the average values of mTOR-Protopanaxatriol, JUN-Protopanaxadiol,TNF-α-ginsenoside Rg1, VEGFA-ginsenoside Rh1, IL-1β-ginsenoside Rh2, STAT3-ginsenoside Rh4 were 58.76678 ± 0.01051nm^2^, in order of 151.97051 ± 0.02124 nm^2^, 82.37012 ± 0.01051, 67 nm^2^, 96,321 ± 0.01178 nm^2^, 76.50828 ± 0.01791 nm^2^, and 101.14167 ± 0.02517 nm^2^ (Fig. 6D). The H-bond interaction for each complex was shown in Fig. 7. The most involved hydrogen bonds during molecular dynamics simulations were IL-1β-Ginsenoside Rh2 (11,454), followed by VEGFA-Ginsenoside Rh1 (10,363), TNF-α-Ginsenoside Rg1 (10,161), STAT3-Ginsenoside Rh4 (10,159), mTOR-Protopanaxatriol (9274), and JUN-Protopanaxadiol (9034). In addition, the IL-1β-Ginsenoside Rh2 and mTOR-Protopanaxatriol systems almost consistently involved hydrogen bonds throughout the molecular dynamics simulations.

### The results of MM-PBSA of ginsenosides and management of diabetes targets

**Figure 8:**
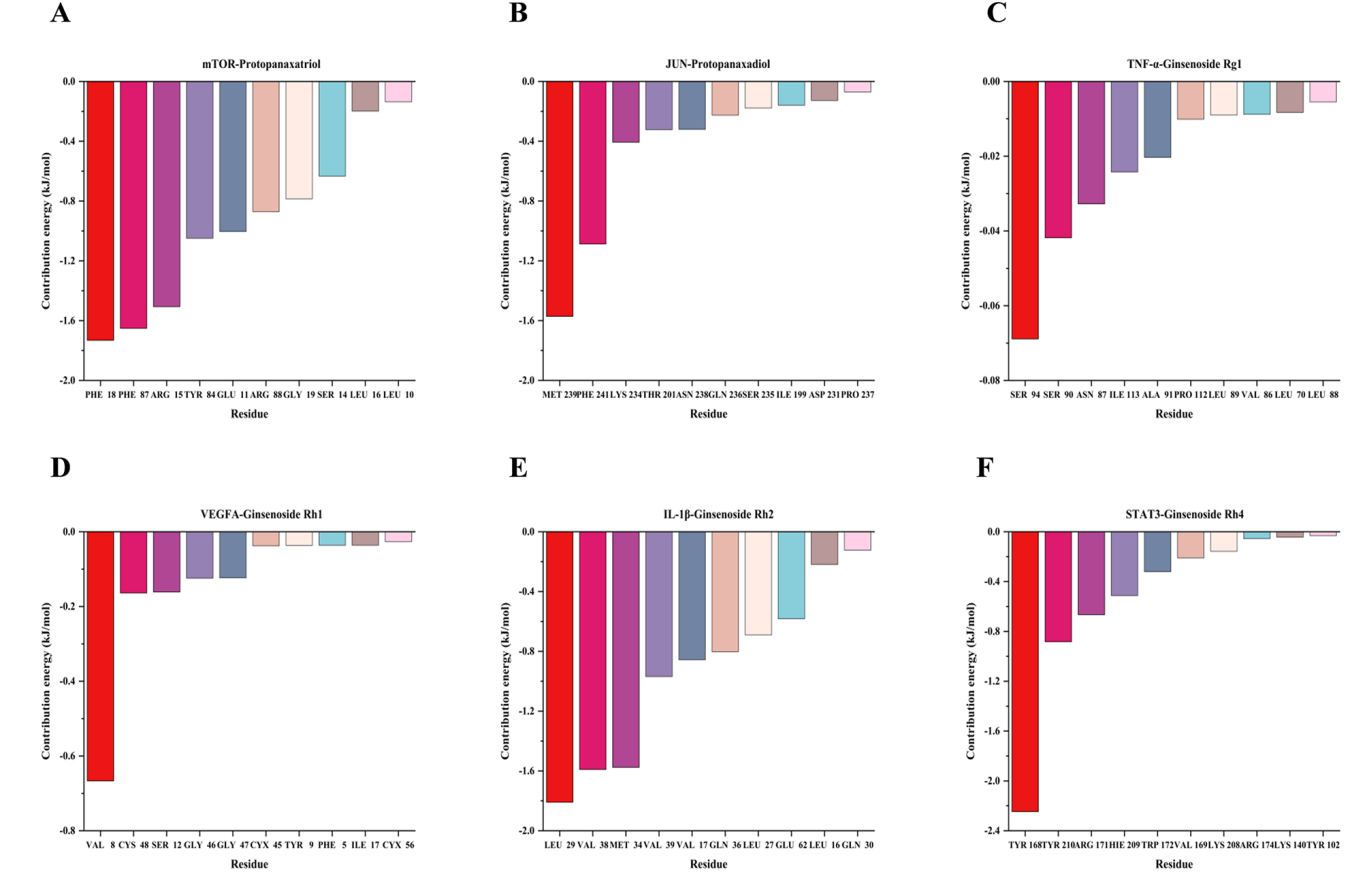
Active site information and contribution values of proteins in the process of protein-molecule binding during molecular dynamics simulation.

The main contribution of amino acid residues in the binding regions of ginsenoside and receptor proteins to the binding free energy is shown in Fig. 8. Six amino acids play an active role in STAT3 and ginsenoside Rh4, IL-1β and ginsenoside Rh2, TNF-α and ginsenoside Rg1, JUN and protopanaxadiol, mTOR and protopanaxatriol, VEGFA and ginsenoside Rh1 complex. These six amino acids and their contribution values were TYR 168 (− 2.24665 kcal/mol), LEU 29 (− 2.24665 kcal/mol), SER 94 (− 0.06888 kcal/mol), MET 239 (− 1.5711 kcal/mol), PHE 18 (− 1.73114 kcal/mol), and VAL 8 (− 0.66648 kcal/mol), respectively. In the complex binding over various small molecule ligands plays a greater contribution.

**Figure 9:**
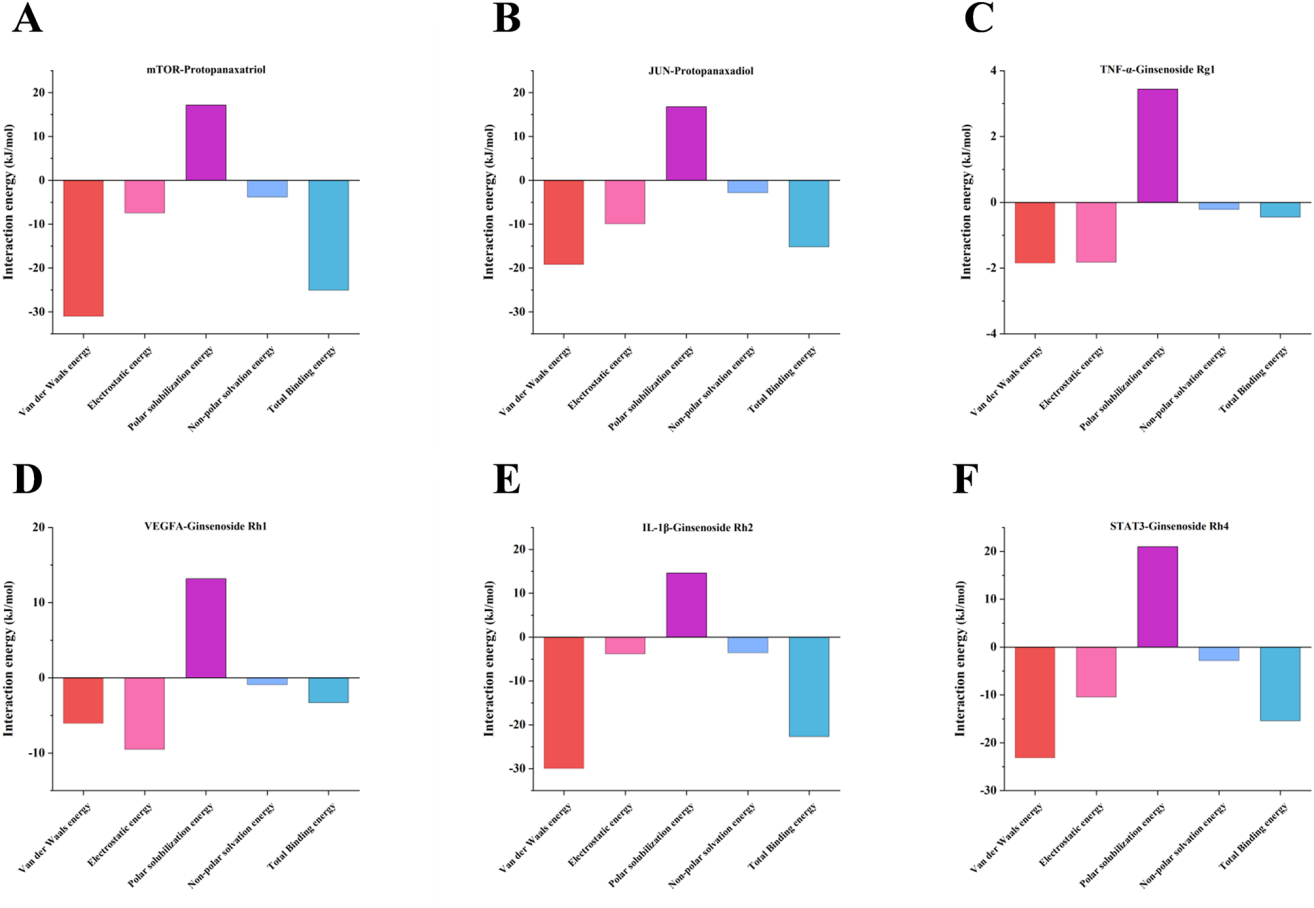
Analysis of the free energy sources involved in the binding of proteins to molecules during molecular dynamics simulations.

The results of the binding free energy analysis shown that the van der Waal energy between mTOR and Protopanaxatriol was − 30.9954 kcal/mol, the electrostatic energy was − 7.4076 kcal/mol, the polar solvation energywas 17.1705 kcal/mol, the non-polar solvation energywas energy was − 3.8093 kcal/mol, and the total binding energy was − 25.0419 kcal/mol (Fig. 9A). The results of the binding free energy analysis shown that the van der Waal energy between JUN and Protopanaxadiol was − 19.2009 kcal/mol, the electrostatic energy was − 9.9003 kcal/mol, the polar solvation energywas 16.7755 kcal/mol, the non-polar solvation energywas energy was − 2.8325 kcal/mol, and the total binding energy was − 15.1582 kcal/mol (Fig. 9B). The results of the binding free energy analysis shown that the van der Waal energy between TNF-α and Ginsenoside Rg1 was − 1.8493 kcal/mol, the electrostatic energy was − 1.8218 kcal/mol, the polar solvation energywas 3.4406 kcal/mol, the non-polar solvation energywas energy was − 0.2152 kcal/mol, and the total binding energy was − 0.4458 kcal/mol (Fig. 9C). The results of the binding free energy analysis shown that the van der Waal energy between VEGFA and Ginsenoside Rh1 was − 6.0465 kcal/mol, the electrostatic energy was − 9.5102 kcal/mol, the polar solvation energywas 13.1659 kcal/mol, the non-polar solvation energywas energy was − 0.9032 kcal/mol, and the total binding energy was − 3.294 kcal/mol (Fig. 9D). The results of the binding free energy analysis shown that the van der Waal energy between IL-1β and Ginsenoside Rh2 was − 29.9513 kcal/mol, the electrostatic energy was − 3.7712 kcal/mol, the polar solvation energywas 14.6004 kcal/mol, the non-polar solvation energywas energy was − 3.5329 kcal/mol, and the total binding energy was − 22.655 kcal/mol (Fig. 9E). The results of the binding free energy analysis shown that the van der Waal energy between STAT3 and Ginsenoside Rh4 was − 23.1377 kcal/mol, the electrostatic energy was − 10.4156 kcal/mol, the polar solvation energywas 20.9973 kcal/mol, the non-polar solvation energywas energy was − 2.8245 kcal/mol, and the total binding energy was − 15.3806 kcal/mol (Fig. 9F). Among the six systems, the mTOR—Protopanaxatriol and IL-1β -Ginsenoside Rh2 systems had the lowest total binding energy and the most stable binding.

## Discussion

DM remains an insurmountable problem worldwide^29^. Chinese herbal medicine has been handed down in China for thousands of years and has a deep historical heritage. A large number of ancient texts on Chinese medicine have clearly documented the effects of herbs to manage DM, such as ginseng^30,31^.

There is increasing evidence that ginsenosides have anti-diabetic and insulin-sensitizing properties. At the same time, ginsenosides not only have the effects of lowering blood sugar, managing insulin sensitivity, and regulating lipid metabolism but also can alleviate the occurrence of DM^32,33^.

Our group conducted some research on ginsenosides to manage DM. The ginsenoside 20(S)-Ginsenoside Rg3 had a protective effect on animal model DM by regulating the MAPK/NF-κB signaling pathway^34^. Rh1 ameliorates DM through AMPK/PI3K/Akt-mediated inflammatory and apoptosis signaling pathways^12^. The malonyl ginsenosides significantly reduced the fasting blood glucose, triglyceride, total cholesterol, low-density lipoprotein cholesterol levels, etc., and managed glucose tolerance and insulin resistance^35,36^. The results showed that the hypoglycemic and insulin-sensitizing capabilities of Compound K (CK) on type 2 DM induced by HFD/STZ via down-regulation of PEPCK and G6Pase expression in the liver^37^. We found that CK could provide beneficial anti-diabetic effects in DM mice, and this protective effect may be mediated by preventing β-cell apoptosis by inhibiting the AMPK-JNK pathway^38^. The predicted results of the present work almost coincide with the findings of our group.

The results of KEGG signalling pathway enrichment analysis indicated that the above signalling pathway might be a significant signalling pathway for ginsenosides to manage DM. However, whether the unreported saponins in ginseng can manage DM mellitus and its mechanism of action are still unclear. Therefore, this study aimed to investigate the managing effects of ginsenosides on DM and their possible mechanisms of action based on network pharmacology and the molecular docking methodology.

As seen above, ginsenosides, as the main active ingredient, can be effective against DM. These previous reports provide sufficient support for our next step to explore the molecular mechanism of components obtained from ginseng to manage DM. In this work, the ginsenosides and their action targets were collected by data mining. In the Venn diagram of the intersection of components in ginseng and DM, 99 cross targets of protein–protein, and 9 core targets were screened by Cytoscape 3.7.0 software. VEGFA plays a major role in endothelial cell growth and angiogenesis and is an essential factor. There were considerable evidence that when DM occurs it often lead to overexpression of VEGFA, which can have a detrimental effect on the body^39,40^. TNF is an important pro-inflammatory factor that can manage DM by reducing insulin signaling through phosphorylation of serine^41^. Moreover, TNF-α is closely associated with diabetic nephropathy and vascular dysfunction^42^.

The results of BP results showed that the ginsenosides were related to the process of positive regulation of protein STK activity, positive regulation of MAP kinase activity, regulation of lipid metabolic process, and positive regulation of reactive oxygen species metabolic process, etc. A lot of evidence has shown that oxidative stress damage leads to lipid peroxidation. Lipid peroxidation causes the rearrangement of peroxyl radicals which can lead to a large of pathological changes in the body. The occurrence of DM often leads to an abnormally high expression of reactive oxygen species (ROS). This can accelerate the onset of cardiovascular disease in DM. Many studies have confirmed that during the development of DM, a shift in glycolytic metabolism occurs, which lead to an overproduction of ROS in monocytes and macrophages. Furthermore, macrophages were produced in large numbers when DM occurs, releasing excessive amounts of pro-inflammatory factors and proteases that lead to inflammation. Because ROS were important mediators in the activation of pro-inflammatory signaling pathways, obesity- and hyperglycemia-induced ROS overproduction may favor the induction of M1-like pro-inflammatory macrophages during the onset and progression of DM^43,44^. This will lead to further deterioration of the disease. These conditions increased the likelihood of cardiovascular disease in people with DM. When a patient suffers from concomitant cardiovascular disease, it was not conducive to the management of DM, forming a vicious circle^45^. Fortunately, we found that ginsenosides may have a certain regulatory effect on the regulation of blood vessel diameter and vascular process in the circulatory system. When DM occurs, the body's skin healing ability is greatly reduced^46^. BP results show that ginsenosides have a regulatory effect on epithelial cell proliferation. This BP may manage DM-induced skin healing difficulties.

These BP were mainly closely related to the molecular functions of steroid binding, steroid hormone receptor activity, and adrenergic receptor activity. And that mainly occur in the mitochondrial outer membrane, an integral component of the presynaptic membrane, neuronal cell body, etc.

Ginsenosides might be involved in a wide range of BP in managing DM and its complications, and these BP are closely related to a variety of molecular functions and cellular components. Overall, our GO results clearly indicated that ginseng could be used to manage DM by modulating BP to ultimately manage it.

A lot of evidence shown that when DM occurs, it tends to cause a series of symptoms such as obesity, insufficient insulin secretion, inflammation, elevated cholesterol, and hardened blood vessels. Our KEGG enrichment analysis showed that insulin, AGE-RAGE, TNF, AMPK, VEGF, adipocyte factor, HIF-1, and PI3K-Akt signaling pathway might be the major signaling pathways^12,38^. The HIF-1 pathway was a well-known regulator of cellular glucose^47^. When diabetics were exposed to a hypoxic environment, HIF was activated and this led to the release of large amounts of inflammatory factors from damaged organs^48,49^. Diabetic patients often had a condition with elevated pro-inflammatory factors, such as TNF-α. TNF-α mediated inflammation, obesity, and insulin resistance were associated with DM^50^. It had been reported that the PI3K/AKT signalling pathway is activated when HKC cells were exposed to a high glucose environment. This promoted islet cell proliferation^51^. Ultimately it was beneficial for DM to manage. Overall, our results suggested that ginsenosides could act on multiple targets and in multiple pathways.

Our findings showed that ginsenosides might regulate the above signaling pathways by acting on Caspase 3, MTOR, VEGFA, MAPK1, JUN, TNF-α, STAT3, IL-1β, EGFR and other targets, thereby managing DM^52,53^. When the binding energy of the receptor protein and the small molecule fraction is less than 0 kcal/mol, it indicates that the two can bind together, and when the binding energy is less than − 7.0 kcal/mol, it indicates that the two can bind together more stably^19^. Our results show 224 molecular dockings with binding energies less than − 7.0 kmol/mol, which accounts for 89.2% of all docking results. This showed that the ginsenosides in ginseng could bind well to the nine core genes. Ginsenosides bind to core targets in a variety of ways, mainly hydrogen bonds, as we know that the binding energy of molecular docking is determined by both the receptor protein and the small molecule ligand. However, it is not clear which of the two has a greater impact on binding energy. In this part of the results, we found that receptor proteins had the greatest effect on the binding energy. The conformation and relative molecular mass of small molecules such as S- or R-type receptors had no significant effect on the binding energy. Although further investigation is needed to obtain more reliable conclusions, these results give us an important hint. Combined with the results of molecular docking implied that ginsenosides have some targeting to the receptor protein. To some extent, it can explain that ginsenosides can manage DM with certain targeting properties.

Furthermore, molecular dynamics simulation, one of the effective tools used to check the stability of the protein ligand complex, was applied to assess the stability of ginsenosides to receptor proteins in the binding pocket in 100 ns. Interestingly, the molecular dynamics simulation results were in agreement with the molecular docking results and suggest the favorable stability along with proper interaction during the time, for both bioactive ingredients with the receptor proteins. The MM-PBSA analysis further clarified the major amino acid sites of different targets and their contribution values. The composition of free energy of ginsenosides and targets during the binding process was also defined clearly. These results further provide that ginsenosides have activity in the management of diabetes mellitus.

So far, more than 100 ginsenosides have been identified. Our prediction results indicated that 28 saponins had an anti-diabetic effect^54^. The predicted amount of saponins accounted for about one fourth of the total. The remaining unpredicted ginsenosides may also have anti-diabetic effects. The reason for this could be the low OB and DL of other ginsenosides or their unclear target. However, compared with the existing reports, our predicted ginsenosides are relatively comprehensive and the mechanism of action is relatively systematic. Meanwhile, ginseng polysaccharide components have been shown to manage DM, but due to some limitations, these components could not be analyzed by network pharmacology^55^. This type of active ingredient still needs further research. Overall, the application of network pharmacology allowed the mechanisms of action of traditional Chinese medicine in the treatment of disease to be explored at the molecular level. It was more systematic to provide valid evidence and basis for the management of DM by ginseng. However, there were shortcomings, such as lesser reported herbs not being searchable, less comprehensive targets for some ingredients, and less software available to correspond to them.

In conclusion, our findings clearly indicated that the managed results of 28 ginsenosides in ginseng on DM were through a multi-component, multi-target, multi-pathway overall regulation. The process involved 99 relevant targets, 2238 GO terms and 169 signaling pathways. This result was consistent with traditional chinese medicine theory. Among the 28 ginsenosides, PPT, Rh1, Rh2, Rh4, Ro, Rg1, and PPD interacted with more targets, BP, and signaling pathways. We speculated that these seven saponins might play a significant role in managing DM. A comparison with the literature, molecular docking, and molecular dynamics simulation showed the authenticity and reliability of our results.

## Supplementary Information

Supplementary Figure S1.

Supplementary Figure S2.

Supplementary Table S1.

Supplementary Table S2.

Supplementary Table S3.

Supplementary Table S4.

Supplementary Table S5.

Supplementary Table S6.

Supplementary Table S7.

Supplementary Table S8.

Supplementary Table S9.

## Data Availability

The datasets of the current study are available in public database from TCMSP (https://www.tcmsp-e.com/tcmsp.php), PubChem (https://pubchem.ncbi.nlm.nih.gov), Swiss Target Prediction (http://www.swisstargetprediction.ch/), UniProt (https://www.uniprot.org/), GeneCards (https://www.genecards.org/), OMIM (https://www.omim.org/), PharmGkb (https://www.pharmgkb.org), TTD (http://db.idrblab.net/ttd/), Drug Bank (https://go.drug/bank.com/), BioLadder (https://www.bioladder.cn/web/#/pro/index), STRING (https://string-db.org/), and PDB (https://www.rcsb.org/). Supplementary files are provided for ingredient targets, disease targets, intersection targets, all GO information, 15 selected GO information, all KEGG information, 15 selected KEGG information, grid box size and center details’ information. These information can be obtained from https://www.scidb.cn/s/2yeMf2.

## Abbreviations

BP: Biological processes
BC: Betweenness centrality
CC: Closeness centrality
CC: Cellular components
CK: Compound K
DM: Diabetes mellitus
DL: Drug likelihood
DC: Degree count
EC: Eigenvector centrality
GO: Gene ontology
KEGG: Kyoto encyclopedia of genes and genomes
LAC: Local average connectivity-based
MF: Molecular function
MM-PBSA: Molecular mechanics Poisson–Boltzmann surface area
NC: Network centrality
OB: Bioavailability
PPT: Protopanaxatriol
PPD: 20(R)-Protopanaxadiol
PPI: Protein–protein interaction
Rh1: 20(S)-Ginsenoside Rh1
Rh2: 20(S)-Ginsenoside Rh2
Rg1: Ginsenoside Rg1
Rh4: Ginsenoside Rh4
Ro: Ginsenoside Ro
ROS: Reactive oxygen species
STK: Serine/threonine kinase
TCMSP: Traditional Chinese medicine systems pharmacology
