# Supplementary material for: Exploring the mechanism of active components from ginseng to manage diabetes mellitus based on network pharmacology and molecular docking
Source: Sci Rep. 2023 Jan 16;13:793. doi: 10.1038/s41598-023-27540-4 (PMC9842641; doi:10.1038/s41598-023-27540-4)
Supplement: Supplementary file 1 — Supplementary Figure S1. [file 41598_2023_27540_MOESM1_ESM.pdf]

KEGG Database Project
Kanehisa Laboratories
www.kanehisa.jp

Ref: 221179

Permission is granted to Scientific Reports of Springer Nature Ltd to publish both in print and digital under the CC BY 4.0 open access license the following KEGG pathway map images in the article "Exploring the Mechanism of Active Components from Ginseng to Manage Diabetes Mellitus Based on Network Pharmacology and Molecular Docking" written by Wei Li and colleagues:

- VEGF signaling pathway - Homo sapiens (human) (hsa04370)
- B cell receptor signaling pathway - Homo sapiens (human) (hsa04662)
- TNF signaling pathway - Homo sapiens (human) (hsa04668)
- Insulin signaling pathway - Homo sapiens (human) (hsa04910)

subject to the condition that the original source is acknowledged by citing at least one KEGG paper.

Permission granted: Naomi Takahashi

Naomi Takahashi, Kanehisa Laboratories

Date: 22 August 2022

Copyright holder: Kanehisa Laboratories
