# Supplementary figures and images for: Exploring the mechanism of active components from ginseng to manage diabetes mellitus based on network pharmacology and molecular docking
Source: Sci Rep. 2023 Jan 16;13:793. doi: 10.1038/s41598-023-27540-4 (PMC9842641; doi:10.1038/s41598-023-27540-4)

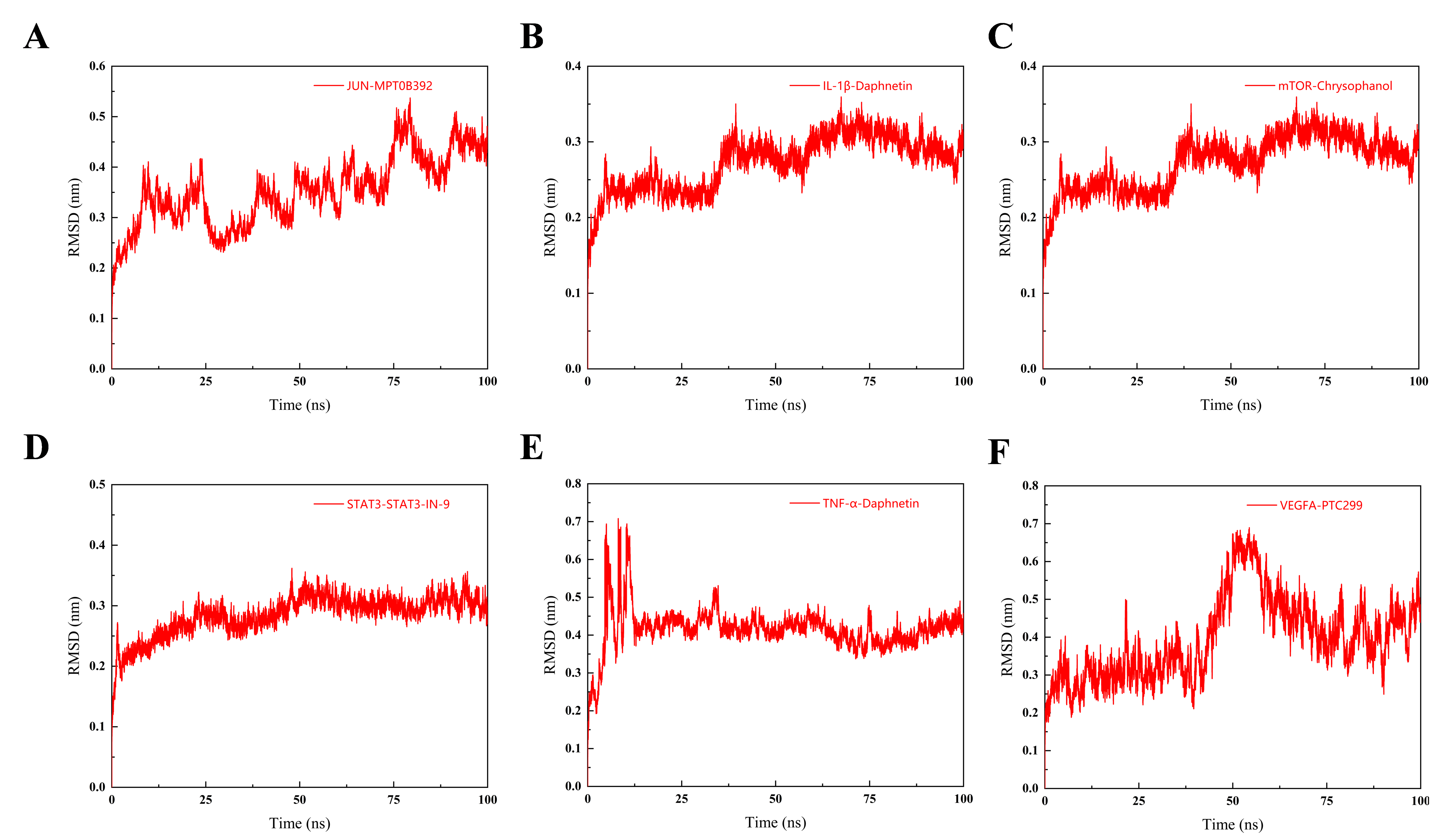

Supplement: Supplementary file 2 — Supplementary Figure S2. [file 41598_2023_27540_MOESM2_ESM.png]
